# Complex pectoral-fin-driven locomotion underlies refined visuomotor behavior in the cichlid *Astatotilapia burtoni*

**DOI:** 10.1016/j.isci.2026.117136

**Published:** 2026-08-18

**Authors:** Nicholas C. Guilbeault, Molly E. Westbrook, Venkatesh S. Krishna, Rida Ansari, Alexander Keth, Aristides B. Arrenberg, Scott A. Juntti, Tod R. Thiele

**Affiliations:** 1 Department of Biological Sciences, University of Toronto Scarborough, Scarborough, ON, Canada; 2 Department of Biology, University of Maryland, College Park, MD, USA; 3 Werner Reichardt Centre for Integrative Neuroscience, University of Tübigen, Tübigen, Germany; 4 Department of Cell and Systems Biology, University of Toronto, Toronto, ON, Canada; 5 Natural Science Division, Pepperdine University, Malibu, CA, USA

**Keywords:** zebrafish, cichlid, optomotor, visual systems, evolution, neural circuits

## Abstract

The optomotor response (OMR) is a conserved visuomotor reflex that stabilizes position during optic flow. The OMR has been extensively studied in zebrafish but examined in detail in only a small number of other fish species. Here, we characterize the OMR in larval cichlids (*Astatotilapia burtoni*). Using high-resolution behavioral assays, we reveal striking species differences in alignment accuracy, speed matching, and swimming kinematics. While zebrafish employ discrete tail-driven swims and stepwise turns, cichlids exhibit continuous swimming, dynamic pivot maneuvers, and backward locomotion powered by complex pectoral fin movements (labriform swimming). Local contrast analyses indicate inverted, species-specific visual feature tuning for the OMR. These results, together with a prior report of similar prey-capture kinematics between these species, highlight the modularity of evolved visuomotor specializations. Together, our results establish larval cichlid optomotor behavior as a tractable system for elucidating how evolutionary history and ecological demands shape neural circuits to produce diverse solutions to a highly conserved visuomotor behavior.

## Highlights

• Cichlids show greater accuracy and speed matching than zebrafish in the larval OMR
• Cichlids employ coordinated pectoral-fin and tail movements when tracking optic flow
• Cichlids uniquely track optomotor stimuli while swimming backward
• Cichlid and zebrafish have inverted preferences for local contrast cues that drive OMR

## Graphical abstract

**Figure:**
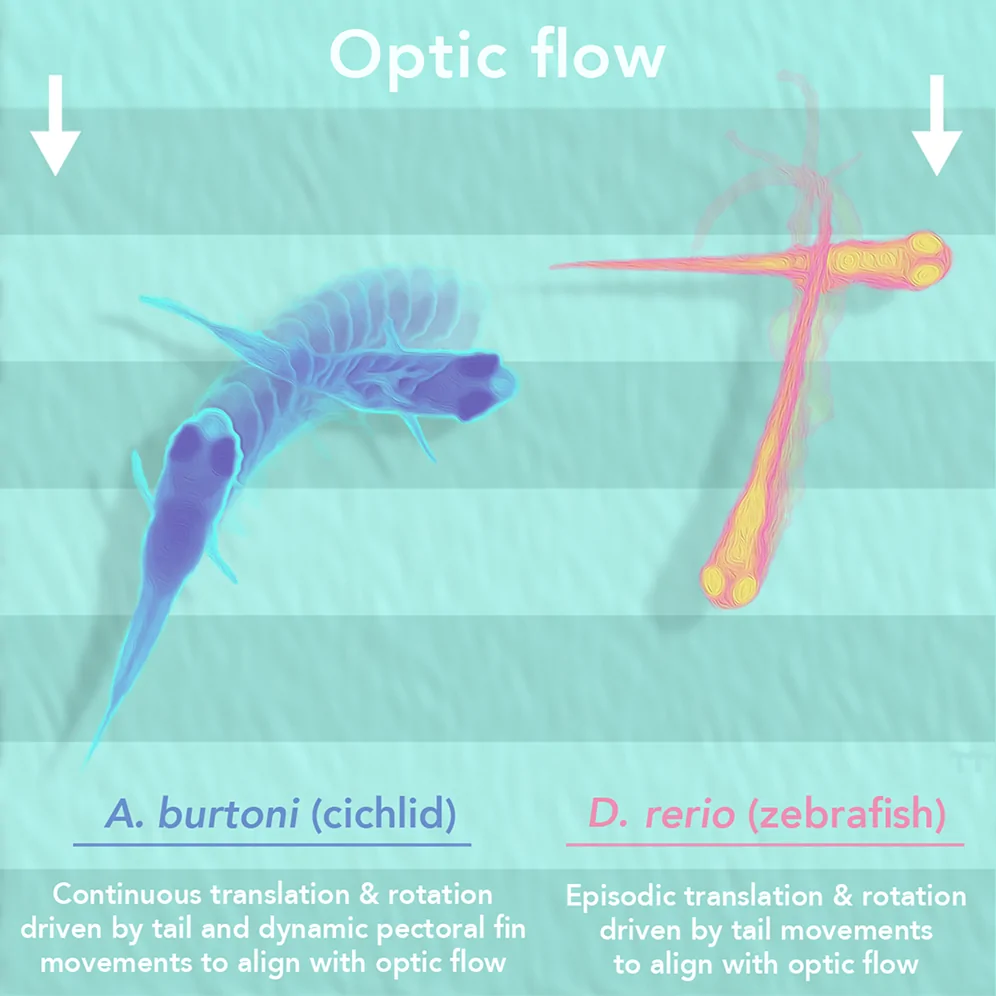

## Teaser

Zoology; Neuroscience; Evolutionary biology

## Introduction

The extraordinary diversity of behavior observed across the animal kingdom is enabled by neural circuit specializations that improve species fitness within ecological niches. Understanding how the nervous system evolves and produces behavioral variation remains a fundamental question for neuroscience and evolutionary biology.^1^^,^^2^^,^^3^^,^^4^ A powerful framework for addressing this question is comparative neurobiology and behavior across related species, grounded in a deep understanding of the habitats and sensory demands in which their behavioral repertoires evolved. This comparative neuroethological approach has been incredibly fruitful for identifying species differences in the genetic and neural mechanisms that subserve behavioral outputs.^3^^,^^5^

The foundational quartet of genetically tractable animal models—*C*. *elegans* (nematode), *Drosophila melanogaster* (fruit fly), *D*. *rerio* (zebrafish), and *M*. *musculus* (mouse)—has increasingly been studied in neuroethological contexts using modern optophysiological tools that target brain function across scales.^6^^,^^7^^,^^8^^,^^9^ However, comparative studies using these technologies in related species remain limited, although this trend is rapidly expanding.^10^^,^^11^^,^^12^^,^^13^ Further insights into brain mechanisms underlying behavioral variation will emerge from expanding the use of genetically encoded systems neuroscience tools across both classical and emerging model species. Recently, research has begun to push the boundaries of genetic tractability using engineering methodologies such as transposon- and CRISPR-based approaches.^14^^,^^15^^,^^16^^,^^17^

The optomotor response (OMR)—body movements elicited by optic flow stimuli—appears to be ubiquitous in animals with eyes.^18^^,^^19^ Despite this ubiquity, the extent to which OMR behavior and its underlying neural mechanisms vary across fish species remains largely unexplored.^13^ The OMR is particularly well characterized in *D*. *rerio*, with a detailed understanding of its behavioral structure and neurobiological underpinnings derived from large-scale neurophysiological recordings.^20^^,^^21^^,^^22^ This extensive body of work provides a broad foundation for identifying divergence in the neural encoding of OMR across teleost species. Such comparisons may illuminate conserved circuit motifs while also uncovering species-specific adaptations. Notable recent examples of this approach include comparisons of swimming and supraspinal motor control between *D*. *rerio* and the related danionin *Danionella cerebrum* (diverged ∼36 Mya), as well as a recent study examining optomotor behavior and associated brain activity in the same species pair.^12^^,^^13^

Here, we provide the first characterization of optomotor behavior in larval *Astatotilapia burtoni*, an African cichlid endemic to Lake Tanganyika and its tributaries, and contrast it with the OMR of *D*. *rerio*. *A*. *burtoni* is a commonly used neuroethological model,^23^^,^^24^ particularly for adult social behavior, with recent work also utilizing larval stages for comparative investigations of prey capture behavior.^25^ CRISPR gene manipulations and insertional transgenesis are well established in this species, enabling modern functional studies of the genetic and neural underpinnings of behavior.^26^^,^^27^^,^^28^ By comparing behaviors of *A*. *burtoni* and *D*. *rerio*, we seek to gain insight into the brain evolution that has shaped these lineages since their last common ancestor ∼220 Mya.^29^ Such divergence provides an opportunity to uncover both conserved features of shared behaviors and species-specific specializations. In this study, we identify differences in OMRs between *A*. *burtoni* and *D*. *rerio* spanning both sensory processing and motor control. Notably, cichlid larvae display intricate pectoral-fin-driven locomotion that enables precise alignment with, and smooth following of, optic flow while facing forward or backward. This work establishes critical groundwork for future investigations into teleost niche-driven brain adaptations that diversify a core visuomotor behavior.

## Results

### Optic flow drives continuous forward and backward swimming in *A*. *burtoni*

**Figure 1:**
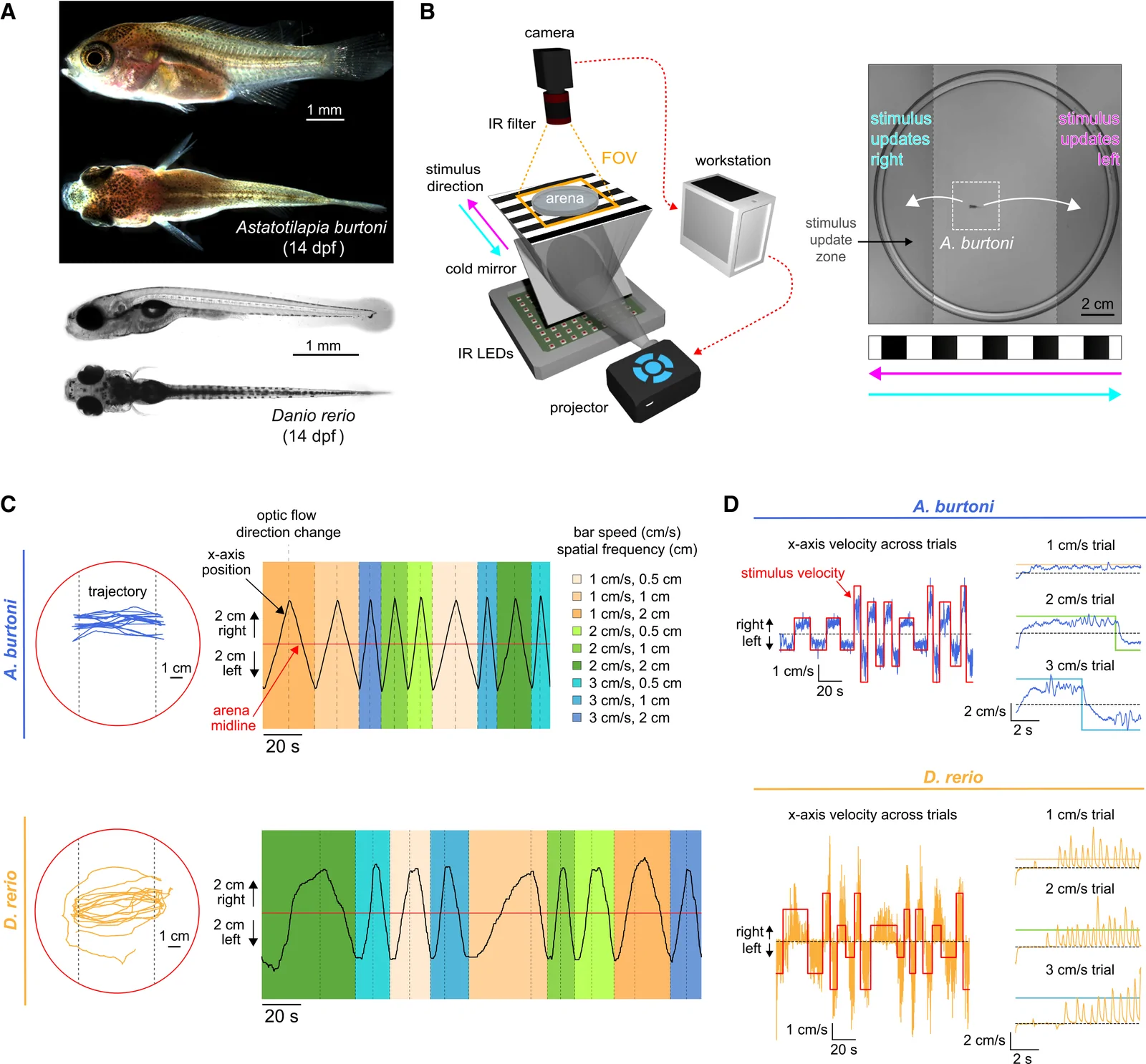
Assay for comparing optomotor performance in *A*. *burtoni* and *D*. *rerio* larvae (A) Morphology of the two species at 14 dpf. (B) Left: schematic of the optomechanical setup used to assess optomotor performance (not to scale). Right: image of a cichlid (white dashed box) within the 15 cm glass dish that serves as the assay arena. Two stimulus update “end zones” (pseudo-shaded dark) sit at the left and right regions of the dish extending 3.75 cm from the dish edge. Fish entering the left end zone trigger a rightward stimulus, while entering the right end zone triggers a leftward stimulus. (C) Left: trajectories of an *A*. *burtoni* larva (top) and *D*. *rerio* larva (bottom) across an entire assay. Right: fish position along the motion direction (*x* axis position) over time (black). Midway point of the arena is depicted as a red line. Completion of a trial is denoted by a vertical gray dashed line. Colors indicate different combinations of grating speed and spatial period. The length of the vertical arrows correspond to 2 cm of movement in the horizontal dimension. (D) Fish velocity along the axis of optic flow across the entire experiment with the stimulus velocity (red line) at three different velocities (1, 2, and 3 cm/s) plotted over time with speed depicted in blue (*A*. *burtoni)* or orange (*D*. *rerio)*. Trials depicted are the same examples as in Figure 1C. At right are high resolution velocity traces for each species at each stimulus speed.

To determine whether the OMR differs between *A*. *burtoni* and *D*. *rerio*, we developed an assay that drove fish back and forth across an arena using motion stimuli with varying parameters. Movements of individual fish were tracked, and entries into left and right “end zones” triggered presentation of a new stimulus in the opposite direction. Gratings of different spatial periods (0.5, 1, 2 cm; corresponding to 0.02, 0.01, 0.008 cycles/degree) were presented at different speeds (1, 2, 3 cm/s), in random sequence (Figures 1B and 1C). Larval *A*. *burtoni* are substantially larger in both length and width than *D*. *rerio*, with a body length roughly twice as long at 14 days post-fertilization (dpf). While *D*. *rerio* begin free swimming at ∼4 dpf, the large yolk of *A*. *burtoni* delays this onset until ∼10 dpf (Figure 1A). To account for species-specific developmental timing, we assessed the OMR in *D*. *rerio* at 7 and 14 dpf and in *A*. *burtoni* between 10 and 15 dpf. No significant age-dependent differences in OMR performance were detected within either species (Figure S1). Comparisons of trajectories from exemplary fish of each species revealed that *A*. *burtoni* can swim with more direct trajectories than *D*. *rerio* and produce sharper directional transitions when stimuli reverse direction (Figure 1C). Strikingly, *A*. *burtoni* were observed to swim backward for extended periods, enabling rapid reversals of swim direction (Videos S1 and S2). Fish could also be observed transitioning smoothly from backward to forward swimming, typically when backward propulsion failed to match the stimulus velocity (Video S2).

Video S1. Optomotor response in *A*. *burtoni* A group of larval cichlids performing the optomotor response to moving gratings in alternating directions. Video acquired at 200 Hz; playback slowed 4x beginning at the 5 s mark

Video S2. Exemplary *A*. *burtoni* during the optomotor performance assay A 15 dpf larval *A*. *burtoni* performing the OMR. Original video was streamed at 200 Hz with 800 x 800 pixel resolution. The video stream was then down sampled to 30 Hz. The visual stimulus was acquired synchronously with the down sampled video by sampling the latest pixel data in the frame buffer at the rate of the downsampled video and overlaying onto the video stream with a 50% opacity

In contrast, *D*. *rerio* did not perform backward swimming, consistent with the lack of previous reports in the extensive optomotor literature for this species.^30^ In lieu of backward swimming, *D*. *rerio* larvae reversed direction with rounded trajectories to realign themselves with the direction of optic flow (Figure 1C and Video S3). Inspection of velocity traces from these fish also identified differences in the continuity of swimming. The *A*. *burtoni* larva remained in constant motion, with its velocity scaling to closely match the stimulus velocity (Figure 1D). This contrasts with *D*. *rerio* larva that swam with discontinuous velocity resulting from the well-characterized bout structure of swimming at early life stages^30^^,^^31^ (Figure 1D). These initial observations revealed stark species differences in optomotor swimming mode and the continuity of self-motion. To our knowledge, this is also the first report of any fish following optic flow through backward swimming. In the following sections, we systematically quantify species differences in OMR performance and identify the distinct kinematics that underlie them.

Video S3. Exemplary *D*. *rerio* during the optomotor performance assay A 14 dpf larval *D*. *rerio* performing the OMR. Original video was streamed at 200 Hz with 800 x 800 pixel resolution. The video stream was then down sampled to 30 Hz. The visual stimulus was acquired synchronously with the down sampled video by sampling the latest pixel data in the frame buffer at the rate of the downsampled video and overlaying onto the video stream with a 50% opacity

### *A*. *burtoni* follow optic flow stimuli with greater accuracy than zebrafish

**Figure 2:**
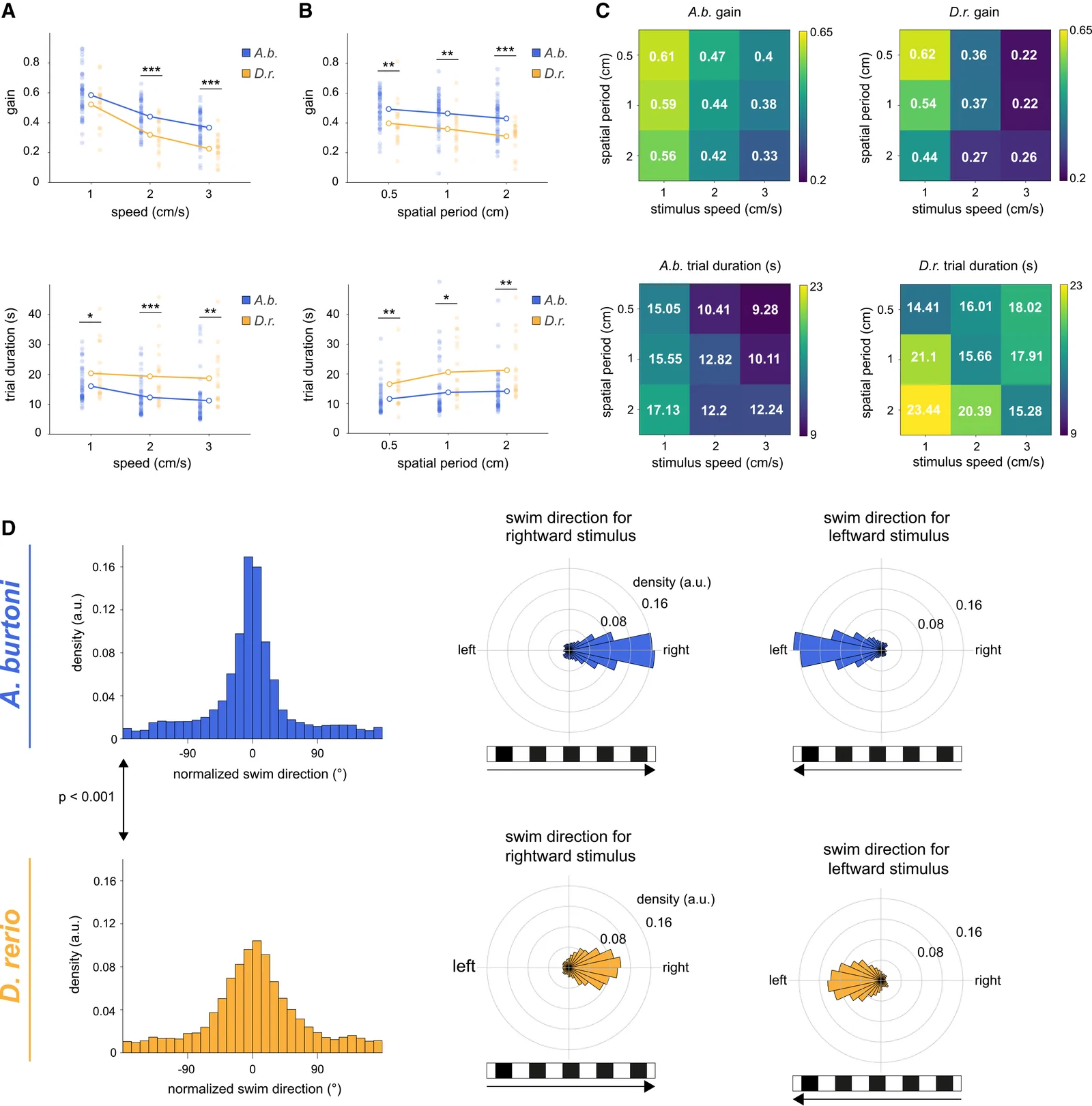
*A*. *burtoni* and *D*. *rerio* exhibit differences in three optomotor response metrics (A) Swim gain (top graph) and trial duration (bottom graph) for each species at each stimulus speed, collapsed across spatial periods. Means are connected by lines; colored circles represent individual fish (*A*.*b*., *n* = 49; *D*.*r*., *n* = 18). Gain (means): 1 cm/s, *A*.*b*. = 0.59, *D*.*r*. = 0.52 (*p* = 0.11, Tukey’s test); 2 cm/s, *A*.*b*. = 0.44, *D*.*r*. = 0.32 (*p* < 0.001); 3 cm/s, *A*.*b*. = 0.37, *D*.*r*. = 0.23 (*p* < 0.001). Trial duration (means): 1 cm/s, *A*.*b*. = 16.06, *D*.*r*. = 20.35 (*p* < 0.05); 2 cm/s, *A*.*b*. = 12.30, *D*.*r*. = 19.36 (*p* < 0.001); 3 cm/s, *A*.*b*. = 11.22, *D*.*r*. = 18.68 (*p* < 0.01). (B) Swim gain (top graph) and trial duration (bottom graph) for each species at each spatial period, collapsed across speed. Gain (means): 0.5 cm, *A*.*b*. = 0.50, *D*.*r*. = 0.40 (*p* < 0.01, Tukey’s test); 1 cm, *A*.*b*. = 0.47, *D*.*r*. = 0.36 (*p* < 0.01); 2 cm, *A*.*b*. = 0.43, *D*.*r*. = 0.31 (*p* < 0.001). Trial duration (means): 0.5 cm, *A*.*b*. = 11.58, *D*.*r*. = 16.56 (*p* < 0.01); 1 cm, *A*.*b*. = 13.81, *D*.*r*. = 20.60 (*p* < 0.05); 2 cm, *A*.*b*. = 14.18, *D*.*r*. = 21.24 (*p* < 0.01). (C) Heatmaps display gain and trial duration values for each stimulus combination. (D) Left: density histograms for each species show normalized swimming direction, with 0° representing perfect alignment to the stimulus. Distributions differ between species (two-sample Kolmogorov-Smirnov test, *p* < 0.001). Right: radial density histogram depicting swimming direction in rightward and leftward stimulus trials. ∗*p* < 0.05, ∗∗*p* < 0.01, ∗∗∗*p* < 0.001.

We assessed each species’ performance in the optomotor task by measuring speed matching to the stimulus (gain), time to complete the assay, and swim alignment with the stimulus direction. Swimming gain was calculated as the ratio between swimming velocity and the stimulus velocity, with a gain of 1 indicating perfect stimulus-speed matching. Nine stimulus speed and spatial frequency combinations were tested twice in back-to-back trials, one in each direction across the dish, resulting in 18 total trials per fish. A three-way mixed ANOVA analysis combining trials from all stimulus parameter combinations (speed and spatial period) uncovered significant main effects of species, stimulus speed, and spatial period (all *p* < 0.001). Combining gain values from all stimulus combinations revealed that *A*. *burtoni* more faithfully matched the stimulus speed compared to *D*. *rerio* across assay conditions (Figure S2). Collapsing across spatial periods uncovered higher gain values for *A*. *burtoni* for speeds ≥2 cm/s (Figures 2A and 2C). Similarly, collapsing across speeds uncovered that *A*. *burtoni* more accurately matched the stimulus speed at all spatial periods (Figures 2B and 2C). We also measured how long it took fish to successfully follow the stimulus between target zones, offering additional insight into optomotor swimming efficiency. For example, a high-gain fish that follows an indirect path across the arena may require more time to complete the trial as a low-gain fish swimming parallel to the stimulus. Using the same analyses as for gain, *A*. *burtoni* completed trials substantially faster than *D*. *rerio* across all stimulus speeds and spatial frequencies, reflecting more efficient visuomotor coordination and streamlined path trajectories (Figures 2A–2C and S2). Together, these analyses show that larval *A*. *burtoni* exhibited greater stimulus speed matching and swimming efficiency than *D*. *rerio* under the tested optomotor conditions, with performance differences most pronounced during stimuli characterized by fast, high-frequency motion, conditions which place greater demands on visuomotor processing.

Performance in an optomotor task can also be assessed by examining how well an animal’s swim direction is aligned to the stimulus direction. To investigate alignment to the stimulus, we calculated swimming direction, normalized to the direction of optic flow across all combinations of stimulus speeds and spatial periods. Comparing the distribution of swim angles showed that *A*. *burtoni* larvae were more closely aligned with the stimulus than *D*. *rerio* (Figure 2D). *A*. *burtoni* swam within ±8.5° of the stimulus direction 27% of the time compared to 13% in zebrafish. Furthermore, cichlids spent less than 49% of the time oriented outside the ±22.5° range relative to the stimulus direction, whereas zebrafish spent approximately 70% of the time outside this range. In summary, results from our analyses of gain, trial duration, and swim direction alignment demonstrate that *A*. *burtoni* larvae outperformed *D*. *rerio* in our optomotor paradigm in their ability to continuously match speed and align to optic flow.

### Temporal engagement in optomotor swimming differs between the species

In our initial observations of *A*. *burtoni* optomotor behavior, fish followed optic flow for several minutes before consistently transitioning to circling along the dish edge, reminiscent of thigmotaxis. To quantify differences in optomotor swimming engagement we used an assay where fish were presented with a continuously moving grating for 15 min (speed: 1 cm/s, spatial period: 1 cm). In confirmation of our initial observations, the assay uncovered pronounced differences in the length of time each species engaged in the optomotor task. *A*. *burtoni* exhibited optomotor swimming comparable to that observed in the previous, optomotor performance assay, before transitioning sharply to persistent circular swimming along the arena wall (Figure S3). In comparison, *D*. *rerio* could engage in continuous optomotor swimming for the full 15-min assay duration (Figure S3). We calculated a thigmotaxis score for each fish as the amount of time spent within 2 cm of the dish edge divided by the total assay time. This analysis confirmed that *A*. *burtoni* spent more time performing thigmotaxis than *D*. *rerio*, with a transition to continuous thigmotactic swimming occurring ∼3 min into the assay (Figure S3). Potential explanations for the delayed increase in thigmotaxis-like behavior include heightened anxiety in *A*. *burtoni* from prolonged assay exposure and/or habituation to the stimulus.

### Optomotor swimming in each species is coupled to distinct local contrast cues

**Figure 3:**
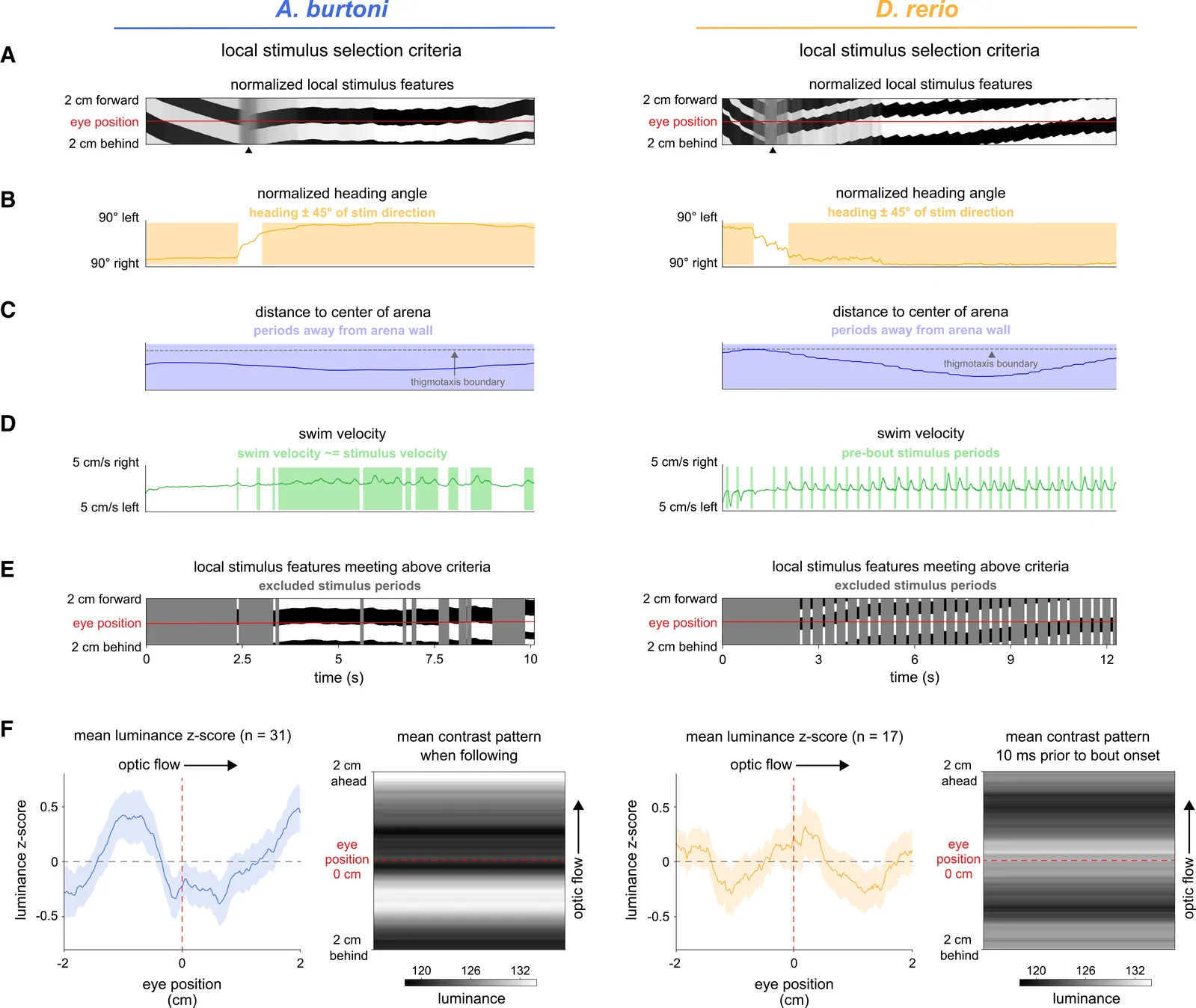
Distinct contrast cues correlate with optomotor swimming across species (A) Stimulus features normalized to the position and heading direction for a single *A*. *burtoni* (left) and *D*. *rerio* (right) 2 cm in front of and behind the eyes (red line). A black arrowhead indicates when the stimulus changed direction. (B) Heading direction along the *x* axis (orange line). Shaded areas (orange) represent periods when the absolute heading angle was ±45° of the absolute stimulus direction. (C) Distance to the arena center over time (blue line). Shaded areas (blue) represent periods when the fish was within the central area of the arena. Thigmotaxis boundary is displayed as a dashed gray line. (D) Swimming velocity along the *x* axis over time (green line). *A*. *burtoni*: shaded areas (green) represent periods when swim velocity exceeded 0.5 cm/s. *D*. *rerio*: shaded areas (green; width not to scale) represent periods 10 ms before the onset of a bout. (E) Resulting set of local stimulus patterns after filtering for the above parameters. (F) Left: mean luminance *Z* score relative to eye position (cm) for *A*. *burtoni* (*n* = 31, blue) and *D*. *rerio* (*n* = 17, orange). Shaded regions represent mean ± SEM. Right: mean contrast pattern ahead of and behind eye position for *A*. *burtoni* while following optomotor stimulus, and for *D*. *rerio* at 100 ms prior to onset of a swim bout during optomotor tracking. The right-side figures render the stimulus luminance features as a two-dimensional contrast pattern by extending luminance values horizontally along the *x* axis.

A prior study determined that the OMR in *D*. *rerio* is driven by a mixture of local and global visual cues.^32^ Using a head-fixed preparation it was determined that a local light to dark transition approaching from behind is the trigger for optomotor swim bouts, with an additional contribution coming from peripheral global motion. Additionally, motion signals caudal to the eye within the lower temporal field were reported to be the strongest triggers for optomotor bouts.^33^ To investigate whether *A*. *burtoni* optomotor movements are also elicited by an approaching local light-to-dark transition, we modified our assay such that fish were presented with bars of random width between 0.25 and 2 cm traveling at a constant speed of 1 cm/s. The assay ran for 5 min with a newly generated set of bar widths presented during each trial. To improve the sensitivity of our analysis, we chose to only analyze local contrast cues at times when fish were deemed to be following the stimulus (Figure 3A). This included periods when the fish’s heading was within ±45° of the stimulus direction and its position was more than 2 cm from the edge of the dish (Figures 3B and 3C). Given that *A*. *burtoni* swim with more constant velocities than *D*. *rerio*, we sampled the stimulus contrast pattern in different ways for each species. For *D*. *rerio*, we sampled the local contrast pattern 10 ms prior to the onset of bouts aligned within ±45° to the direction of stimulus motion (Figure 3D). For *A*. *burtoni*, we instead sampled contrast patterns whenever a fish’s velocity exceeded 0.5 cm/s and its heading was aligned within ±45° of the stimulus direction (Figure 3D). The above selection criteria produced a filtered set of stimulus contrast patterns when fish were deemed to be engaged in optomotor swimming (Figure 3E). Analysis of local stimulus features during periods of engaged optomotor swimming identified non-random structure for both species (Figure 3F). *D*. *rerio* generated optomotor bouts when a dark band approached from behind, consistent with head-fixed results.^32^ In contrast, *A*. *burtoni* optomotor swimming had a strong temporal correlation with a bright band approaching from behind, with the magnitude of the absolute contrast signal being stronger than the dark-band cue uncovered for *D*. *rerio*. In summary, local contrast cues differed starkly between the species, with the signals being approximately inverted.

### Species-specific kinematics drive optomotor swimming

**Figure 4:**
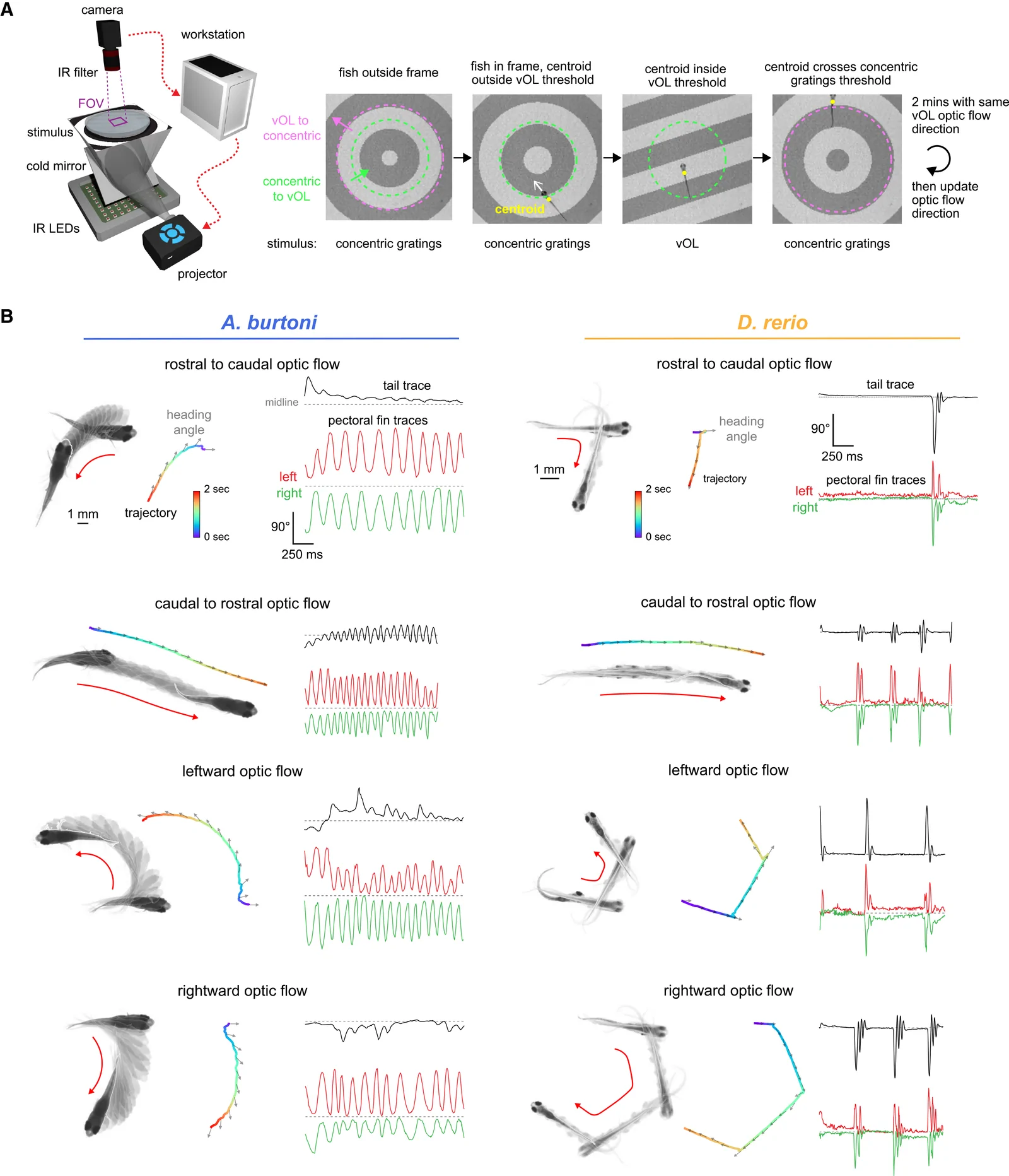
Species-specific tail and pectoral fin kinematics propel optomotor swimming (A) Left: a small field of view (FOV) allows high-resolution tracking of tail and pectoral fins while optomotor gratings are presented in virtual open-loop (vOL). Right: overview of the vOL assay workflow. Fish outside the frame are drawn to the center by converging concentric gratings. When fish enter the frame, software begins to monitor the centroid position and heading angle in real-time. Once fish cross an inner circular boundary (green) a vOL trial commences with optomotor gratings presented in one of four randomly selected directions (forward, backward, leftward, or rightward). Stimuli are locked to the heading direction such that fish cannot escape the directionality of optic flow. The stimulus continues as long as fish remain within a larger secondary boundary (pink). Once fish cross this boundary, concentric gratings again gather fish to the center. The assay workflow runs for 2 min using the same stimulus direction followed by an update to a new randomly selected stimulus direction. (B) Tail and pectoral fin kinematics during each stimulus direction. Recorded video framerate was 332 Hz; example fish image frames are subsampled and displayed at 7 Hz for *A*. *burtoni* and 30 Hz for *D*. *rerio* to aid visualization of swimming trajectories and body movements.

We next examined how each species propelled themselves during optomotor swimming, with particular interest in how *A*. *burtoni* exhibit enhanced maneuverability (notably backward swimming) and maintain continuous velocities. Preliminary observations revealed pronounced kinematic differences, including extensive pectoral fin usage by *A*. *burtoni* (Video S1). To capture both tail and pectoral fin movements during optic flow presentation, we established a high spatial resolution virtual open-loop assay. A concentrically contracting grating was first used to position fish within the imaging field of view, after which one of four optic flow directions was presented: caudal-to-rostral, rostral-to-caudal, leftward, or rightward (Figure 4A). The stimulus was updated in real time to compensate for heading changes, resulting in optic flow that remained constant relative to the fish’s heading (Video S4). Under these conditions, both species produced robust optomotor swimming in the direction of optic flow, consistent with behavioral responses in the OMR performance assay (Figure 4B).

Video S4. Virtual open-loop OMR assay for kinematic tracking Video shows a 15 dpf *A*. *burtoni* performing the OMR during leftward optic flow. A concentric contracting grating gather the fish to the center of the field of view whenever it moves to the periphery. Video was acquired at 332 Hz with 1,088 x 1,088 pixel resolution

**Figure 5:**
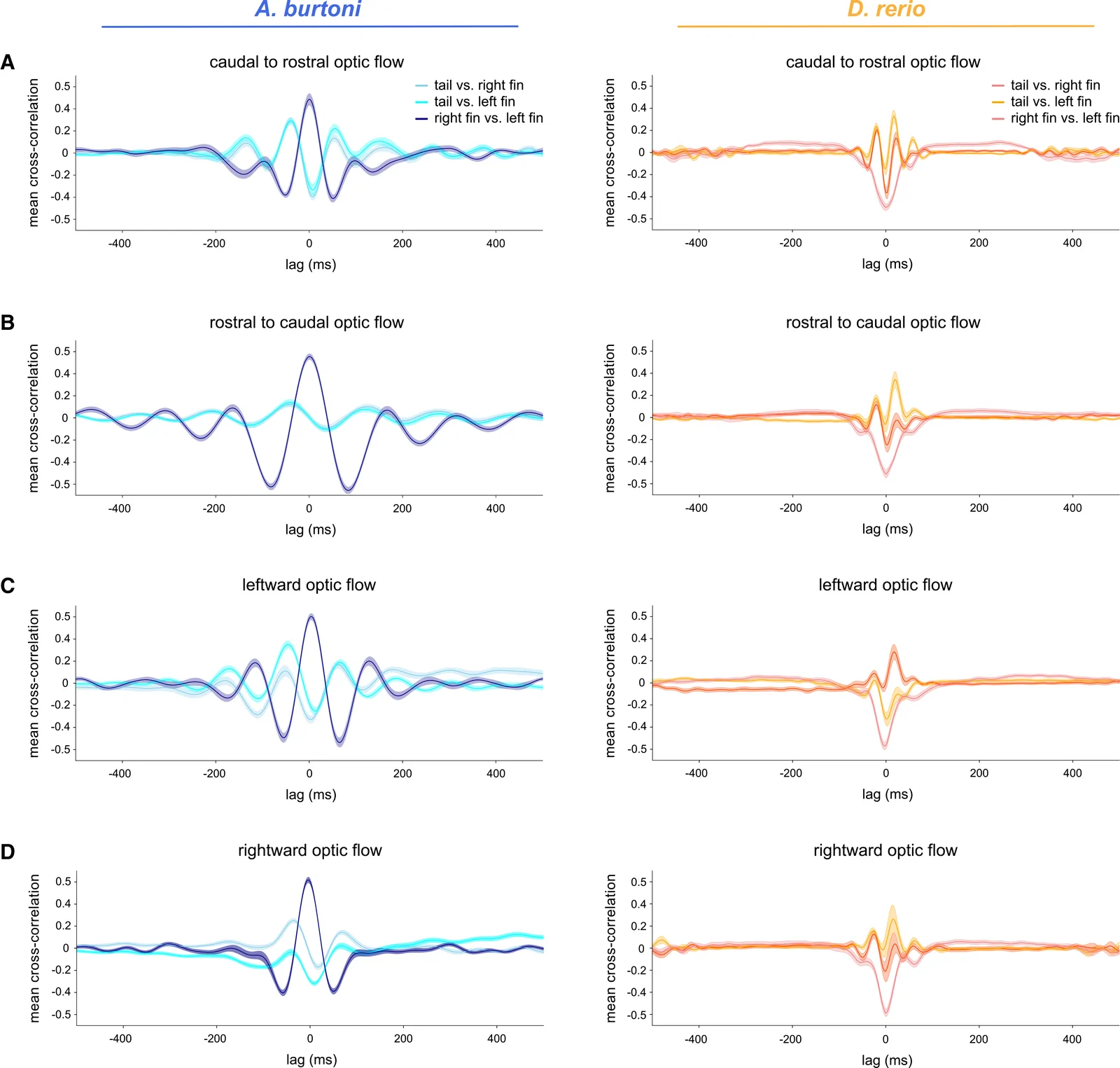
Cross-correlation structure of fin and tail movements during optomotor swimming Mean pairwise cross-correlations between tail and left fin, tail, and right fin, and right fin and left fin are shown for *A*. *burtoni* (left column) and *D*. *rerio* (right column) across four optic flow directions: (A) caudal-to-rostral (forward), (B) rostral-to-caudal (backward), (C) leftward, and (D) rightward. Optic flow stimuli were presented in a virtual open-loop assay in which perceived flow direction was held constant relative to the fish’s heading throughout each stimulus. For each species and condition, pairwise cross-correlations were computed between time series of tail and pectoral fin movements and averaged across individuals. Positive peaks near zero lag indicate temporally coupled movements, while oscillatory profiles at extended lags reflect rhythmic coordination between structures. *A*. *burtoni n* = 4; *D*. *rerio n* = 4. Bands indicate mean ± SEM.

**Figure 6:**
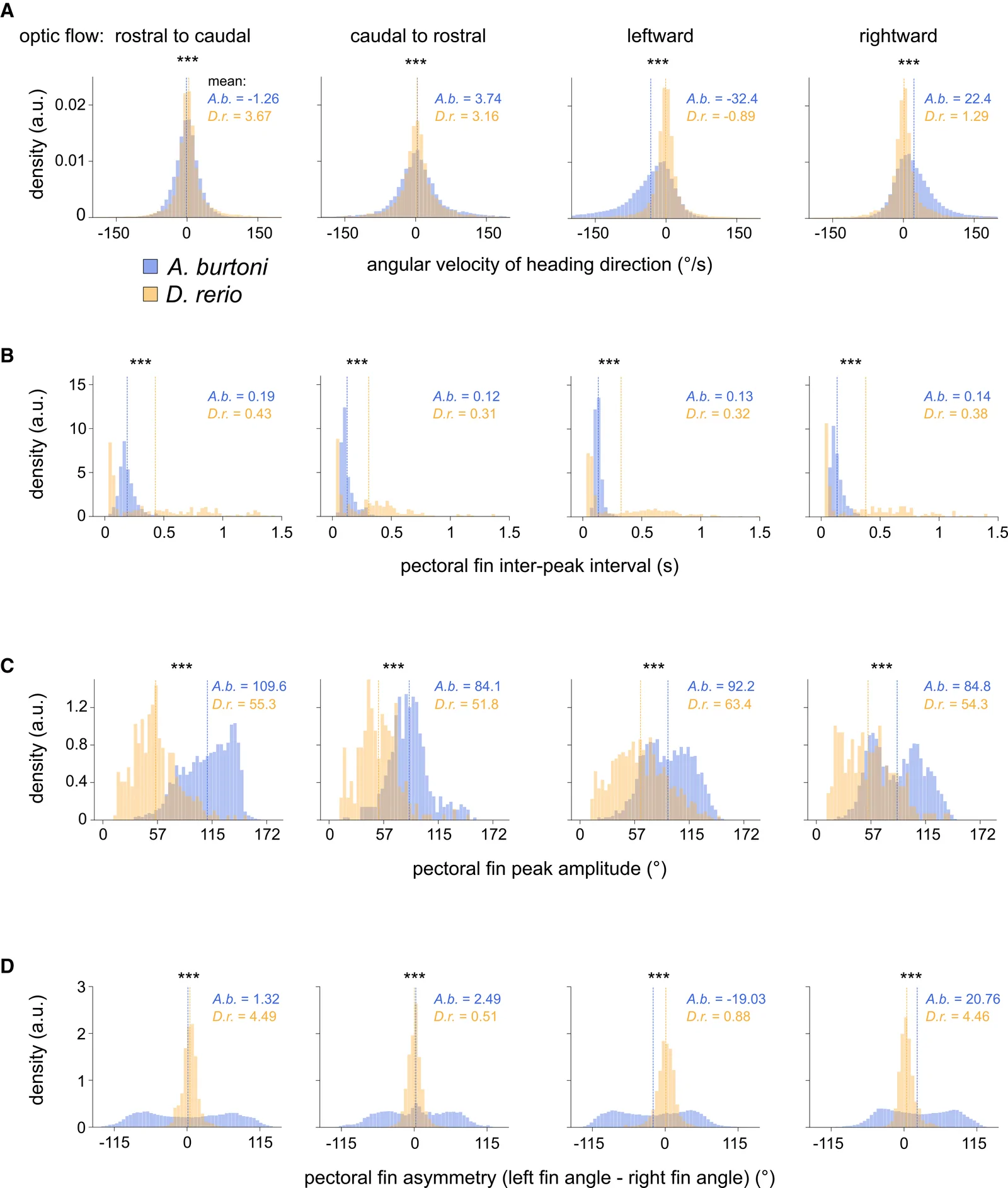
*A*. *burtoni* exhibits dynamic pectoral fin modulation during optomotor swimming (A) Angular velocity of heading direction, calculated by taking the temporal derivative of the heading angle. (B) Time between maximal pectoral fin position; data from both left and right pectoral fins were combined. (C) Absolute pectoral fin peak amplitudes, data from both left and right pectoral fins were combined. (D) Pectoral fin asymmetry calculated as the left fin angle subtracted by the right fin angle. Negative values represent a right fin bias, whereas positive values represent a left fin bias. Vertical dashed lines indicate the mean of each species is displayed. ∗∗∗*p* < 0.001 (two-sample Kolmogorov-Smirnov test). *A*. *burtoni n* = 4; *D*. *rerio n* = 4.

The most pronounced kinematic difference was *A*. *burtoni*’s reliance on pectoral fins to drive locomotion, a mode termed labriform swimming. *A*. *burtoni* pectoral fins moved continuously across all conditions, whereas *D*. *rerio* fin movement was restricted to periods coincident with tail motion. In *A*. *burtoni*, forward-moving gratings typically elicited continuous tail oscillations accompanied by bilaterally antiphase pectoral fin movements, though periods of pectoral fin-driven swimming without tail engagement were also apparent (Figure 4B). This sustained appendage coordination was reflected in cross-correlation structure, which exhibited broad, oscillatory coupling between fins and tail and a strong anti-phase relationship between left and right fins (Figures 5A–5D). In contrast, forward-moving gratings in *D*. *rerio* produced the well-described pattern of discrete swim bouts, each accompanied by 2–3 rapid tail fin beats (Figure 4B). Correspondingly, zebrafish showed narrow, transient cross-correlation peaks centered near zero lag, indicating that fin activity is tightly time-locked to tail-driven bouts rather than generated by an independent rhythmic program (Figures 5A–5D). Zebrafish fin movements were also higher frequency and lower amplitude across conditions, compared to those of cichlids (Figures 6B and 6C).

The greatest divergence in locomotion emerged under rostral-to-caudal optic flow. Backward motion stimuli in *D*. *rerio* elicited large-angle turns with concomitant fin movements, as the fish attempted to incrementally realign with reverse flow that was artificially locked to their heading. By contrast, *A*. *burtoni* executed immediate continuous backward swimming (Figure 4B), powered by alternating high-amplitude pectoral strokes that increased in amplitude over time (Figure 4B). During these strokes, the tail remained relatively straight but often displayed unilateral sustained bends, likely aiding steering to align with reverse flow (Video S5). Cross-correlation analysis revealed that the oscillatory coordination between tail and pectoral fin movements observed during forward swimming was preserved during backward locomotion, but with higher peak cross-correlation values, indicating stronger inter-appendage coupling (Figure 5B).

Video S5. *A*. *burtoni* pose tracking during rostral to caudal optic flow Video shows a 15 dpf *A*. *burtoni* performing the OMR during rostral to caudal optic flow. Video was acquired at 332 Hz with 1,088 x 1,088 pixel resolution. Pose tracking of the pectoral fins and tail was performed using DeepLabCut post data acquisition

During leftward or rightward optomotor stimuli, *D*. *rerio* produced a sequence of turns characterized by large initial tail bends that rapidly shifted the fish’s heading, achieving significantly higher angular velocities than *A*. *burtoni* (Figures 4B and S4). Fin-tail and fin-fin cross-correlations both showed weak, transient coupling near zero lag with little asymmetry between leftward and rightward stimuli (Figures 5C and 5D), consistent with pectoral fins moving briefly in synchrony with tail bouts rather than exhibiting independent or antiphase coordination. *A*. *burtoni* displayed more gradual heading changes resembling banked turns, with angular velocity held roughly constant and both axial and lateral displacement occurring in concert (Figures 4B and 6A). Turning in *A*. *burtoni* involved contributions from both pectoral fins and tail, with fin-tail cross-correlations differing between leftward and rightward optic flow conditions (Figures 5C and 5D), indicating that appendage coordination was modulated according to turn direction. This directional modulation was accompanied by intermittent unilateral tail deflections toward the turn direction that likely aid in rotating the animal (Figure 4B). Together, these findings reveal that *A*. *burtoni* and *D*. *rerio* achieve optomotor stabilization through fundamentally distinct locomotor programs.

## Discussion

Our comparative analysis of the larval OMR in *A*. *burtoni* and *D*. *rerio* uncovered pronounced species differences in optic flow alignment accuracy, speed matching, and behavioral duration. We also found that different contrast patterns evoke optomotor movements in each species and that cichlids exhibited more complex and continuous appendage coordination during propulsion. While cichlids exhibited more refined optomotor performance under our assay conditions, these results should be interpreted within the context of the specific visual and kinematic parameters tested, which may favor species from habitats with specific sensory features. Although the assay conditions were designed to isolate overt behavioral differences, they did not capture the complexity or variability of natural visual scenes. Despite these constraints, the results of this study point to several key differences in how each species processes optic flow and provide a basis for future investigations into how sensory signals are translated into stabilizing movements.

The most prominent species divergence we observed was in locomotor mode. *D*. *rerio* used a combination of forward swimming bouts and stepwise turns to align with optic flow, consistent with numerous previous studies.^30^ Short bursts of pectoral fin movements were confined to periods when the tail was oscillating. In contrast, cichlid larvae swam continuously, employing tail oscillations along with dynamic pectoral fin movements that allowed for smooth translation and heading modulation. While pectoral fin movements typically accompanied tail oscillations, we also observed periods of pectoral fin-only locomotion. Notably, *A*. *burtoni* could generate prolonged stretches of backward swimming powered solely by pectoral fin strokes with a reversed power phase. The most complex motor sequence we observed was a seamless reorientation from backward to forward swimming, likely engaged when fish required additional thrust from the tail to match faster optic flow.

Extensive use of pectoral fins for propulsion is common among members of the large Percomorpha clade of ray-finned fishes, which includes many reef-associated families as well as the Cichlidae.^34^^,^^35^ The labriform swimming we identify at 14 dpf in *A*. *burtoni* is, to our knowledge, the earliest age reported for this complex pectoral fin driven translation and reorientation. Labriform propulsion has not been observed in zebrafish, where pectoral fins primarily regulate pitch and generate water flow across the skin and gills prior to full gill development.^36^^,^^37^ This species difference presents a unique opportunity to compare the sensorimotor control underlying labriform propulsion. Recent advances in understanding the central pattern generator circuits that drive pectoral fin movements in zebrafish provide a foundation for investigating how more complex fin control arises in *A*. *burtoni*.^38^

Zebrafish optomotor behavior has been dissected in great detail at both sensory and motor levels.^39^ Direction-selective retinal ganglion cells (DSGCs) transmit global motion signals primarily to arborization field 5 (AF5) in the forebrain and AF10 in the midbrain, innervated by neurons in the pretectum and optic tectum, respectively.^40^^,^^41^^,^^42^^,^^43^ Although both regions process optic flow, ablation and optogenetic studies indicate that the pretectum is the primary sensory region underlying the OMR.^42^^,^^43^^,^^44^ Little is known about the neural basis of motion processing in *A*. *burtoni*, but given the extensive neural homologies across teleosts,^45^^,^^46^ the overall structure and functionality of visual circuits is likely similar to *D*. *rerio*.^15^^,^^47^^,^^48^ *A*. *burtoni*’s enhanced OMR performance at high spatial frequencies may reflect a greater number of retinal ganglion cells within their substantially larger larval eye, approximately twice the diameter of *D*. *rerio* (Figure 1A). Greater eye size and RGC number likely enhances spatial resolution, enabling extraction of finer details from environmental optic flow. *A*. *burtoni* invest more maternal energy in each offspring, reflected in smaller brood sizes (30–80 embryos) with large yolks (∼3 mm),^15^ which may support enhanced development of retinal and neural structures during larval stages and enable more refined sensory mapping and motor control.

Species differences extend beyond sensory processing to the motor circuits that translate optic flow into locomotion. Pretectal output in zebrafish ultimately converges upon reticulospinal neurons in the midbrain and hindbrain, which in turn activate spinal central pattern generators that coordinate tail movements for optomotor swimming.^49^^,^^50^^,^^51^ In zebrafish, pectoral fin movements during the OMR were tightly coupled to tail bouts, suggesting that fin and tail central pattern generators may be closely coupled. The extensive and asymmetric pectoral fin recruitment observed in *A*. *burtoni* suggests that motor pathways for the tail and fins can be differentially engaged, with pectoral fin CPGs playing a more autonomous role in locomotor control.

The sensory and motor divergences we identify raise the question of what ecological pressures drove these species differences. The visual characteristics of the habitats occupied by these species differ considerably, shaped by differences in substrate, turbidity, and current dynamics.^52^^,^^53^^,^^54^^,^^55^^,^^56^^,^^57^ *A*. *burtoni* inhabit swamps and rivers feeding Lake Tanganyika as well as rocky littoral zones along the lake’s shores, environments that are wave-exposed and hydrodynamically complex.^23^^,^^58^ Substantial wave activity on one of Earth’s largest lakes creates unsteady flow conditions that demand precise stabilizing movements.^59^^,^^60^ Lake Tanganyika also has exceptionally clear water for much of the year,^60^^,^^61^ whereas many zebrafish habitats are relatively turbid, in part due to runoff during seasonal monsoons.^52^^,^^53^^,^^54^^,^^62^ Variation in substrate and water clarity produces distinct spatiotemporal visual environments, likely creating unique selective pressures on sensory systems in each species.^54^ *A*. *burtoni* and its ancestors evolved in varied and dynamic environments, suggesting habitat complexity may have selected for more elaborate visuomotor responses.^63^

These ecological differences may be reflected in the distinct visual features that drive optomotor swimming in each species. *A*. *burtoni* followed dark-to-light transitions approaching from behind, while *D*. *rerio* responded to light-to-dark transitions. A forward-moving OFF edge is known to trigger optomotor swims in *D*. *rerio* and is associated with widespread brain activity tuned to this feature.^32^ The strong local ON edge detected just behind the eye in *A*. *burtoni* during the OMR may reflect differences in retinal ganglion cell tuning or downstream pretectal weighting, potentially driven by ecological environments where high-contrast gradients are more prominent. However, the inverted contrast preferences need not reflect differences at the level of sensory tuning per se; they could equally arise from species differences in how downstream premotor circuit weight or gate incoming visual signals. Labriform propulsion is a common locomotor strategy in fishes from clear, hydrodynamically complex habitats such as coral reefs,^34^ suggesting that the elaborate pectoral fin usage observed in *A*. *burtoni* may reflect adaptation to similar environmental demands. Pectoral fins are also actively used during territorial disputes between males, in which they swim forward and backward while establishing boundaries,^23^ and these motor pathways may similarly be utilized for maternal recall of free-swimming larvae.^64^

While recent work has compared zebrafish with its closer relative *D*. *cerebrum* (diverged ∼36 million years ago) to probe the neural basis of behavioral variation, our study extends this approach to a deeper evolutionary split. *A*. *burtoni* and *D*. *rerio* lineages diverged roughly 220 million years ago,^29^ providing a wider window into how core visuomotor behaviors can evolve and diversify. Consistent with this expectation, we find behavioral differences substantially larger than those reported between *D*. *rerio* and *D*. *cerebrum*.^13^ Like *D*. *cerebrum*, *A*. *burtoni* displays continuous swimming rather than the saltatory style of *D*. *rerio*.^12^^,^^13^ However, *A*. *burtoni* is the only species among the three capable of smooth rotations, and backward swimming via specialized pectoral-fin use. The deep divergence between Cypriniformes (zebrafish) and Cichliformes (cichlids) may ultimately make differences in the neural mechanisms underlying behavior more salient. Nevertheless, a recent prey-capture comparison between *A*. *burtoni* and *D*. *rerio* revealed mostly matching behavioral strategies, with similar eye and tail kinematics, suggesting further commonalities at the neurobiological level. Taken together with our results, these findings reveal how one behavior (prey capture) can remain conserved in a presumed ancestral state, while another (the OMR) undergoes profound evolutionary change.

Future work should explore the retinofugal pathways in *A*. *burtoni* to determine how motion information is routed to and processed by pretectal and tectal targets. Likewise, we predict that descending motor control of the pectoral fins in *A*. *burtoni* involves more complex integration of sensory and premotor inputs, enabling fine modulation during turning and backward swimming. Another intriguing area to investigate is olivocerebellar sensorimotor mismatch processing which likely contributes to the smooth switching observed between backward and forward locomotion when animals sense the need for greater propulsion to stabilize optic flow.^65^^,^^66^ With an increasing number of genetic tools becoming available in *A*. *burtoni*, including CRISPR, transgenesis, Gal4/UAS systems, and GCaMP calcium imaging, the neurobiological underpinnings of visuomotor circuit specializations in this species are increasingly accessible.^15^^,^^27^ Our work reveals the refined sensorimotor capabilities of a larval cichlid and provides a framework for investigating how ecology and evolution diversify the neural circuits underlying a common visuomotor behavior.

### Limitations of the study

The differences in stimuli that drive the OMR suggests that *A*. *burtoni* and *D*. *rerio* prioritize visual information differently to guide optic flow, potentially reflecting adaptations to their native visual environments. However, as our study does not directly observe or manipulate neural systems, we cannot distinguish whether these species extract distinct features from the environment or initiate motor outputs in different contexts. Our study attempted to align these species’ developmental stages, but due to fundamental differences in developmental rate and sizes, it is impossible to perfectly match animals on all dimensions. Our work relies on responses to laboratory-generated visual stimuli, but future experiments using more naturalistic stimuli are required to determine how features of the native habitat activate motor responses. Our results are constrained to studies of OMRs, as we did not assess other behaviors such as prey capture or escape.

## Resource availability

### Lead contact

Further information and requests for resources and reagents should be directed to and will be fulfilled by the lead contact, Tod R. Thiele (todthiele@gmail.com).

### Materials availability

This study did not generate new unique reagents.

### Data and code availability

• All data reported in this study will be shared by the lead contact upon request.
• All original code has been deposited at Zenodo and is publicly available at https://doi.org/10.5281/zenodo.21463359.
• Any additional information required to reanalyze the data reported in this study is available from the lead contact upon request.

## Acknowledgments

This research was supported by an NSERC doctoral scholarship (N.C.G.), NSERC Discovery grant (T.R.T.), a Human Frontier Science Program grant (RGY0079/201, T.R.T., S.A.J., A.B.A., and Emily Cooper), the Canadian Foundation for Innovation and the Ontario Research Fund (T.R.T.), and NIH (R35GM142872, S.A.J.).

## Author contributions

S.A.J., T.R.T., and A.B.A. conceived of the project. N.C.G., M.E.W., A.K., R.A., and V.S.K. designed experiments and collected data. N.C.G. analyzed the data. N.C.G., T.R.T., and S.A.J. wrote the study with comments provided by all co-authors. T.R.T., S.A.J., and A.B.A. provided funding for the project.

## Declaration of interests

Elsevier is the current employer of Rida Ansari and peer review was fully independent of said author.

## Declaration of generative AI and AI-assisted technologies in the writing process

During the preparation of this work the authors used Claude (Anthropic) to assist with editing text during manuscript preparation. After using this tool/service, the authors reviewed and edited the content as needed and take full responsibility for the content of the publication.

## STAR★Methods

### Key resources table

**Table:** 

REAGENT or RESOURCE	SOURCE	IDENTIFIER
**Experimental models: Organisms/strains**		

*Astatotilapia burtoni*	Fernald lab strain, University of Maryland	N/A
Danio rerio	TL strain	N/A

**Software and algorithms**		

BonZeb		https://doi.org/10.1038/s41598-021-85896-x
DeepLabCut		2.2.1.1
Python		3.10.6
NumPy		1.23.3
Pandas		1.5.2
SciPy		1.9.1
Pingouin		0.5.2
Statsmodels		0.13.2
OpenCV		4.6.0.66
All other code developed for this manuscript		https://doi.org/10.5281/zenodo.21463359

### Experimental model and study participant details

Mixed-sex groups of adult *Astatotilapia burtoni*, derived from Lake Tanganyika,^58^ were maintained at the University of Maryland and the University of Toronto Scarborough. Cichlids were maintained in 32- to 120-L community tanks with pH ∼8.0, 25°C water temperature, and a 12:12-h light:dark cycle with 15-min dusk and dawn periods. Gravel and terracotta pots provided substrate and territories for cichlid reproduction. Cichlids were fed daily with cichlid food flakes (Jehmco) between 0900 h and 1100 h. Zebrafish were raised and bred at 28°C on a 14 h light/10 h dark cycle at the University of Toronto Scarborough. Larvae were raised in 10 cm petri dishes in group sizes of 30 or less and fed paramecia starting from 4 dpf and ending on 7 dpf. After 7 dpf, zebrafish were housed in 1.2 L tanks with system water and fed GEMMA Micro dry food (Skretting). All animal maintenance and experimental procedures undertaken at the University of Maryland were approved by the Institutional Animal Care and Use Committee of the University of Maryland (protocol 2133862). All experiments at the University of Toronto were approved by the University of Toronto Local Animal Care Committee and adhered to the governance of the Canadian Council on Animal Care (protocols 20012556, 20011546). As sexual dimorphisms are not apparent at these early stages, sex of fish was not taken into account. To account for species differences in the age of onset of free-swimming behavior, zebrafish were tested at both 7 and 14 days post-fertilization (dpf), whereas cichlids were tested between 10 and 15 dpf. The study was carried out in accordance with the ARRIVE guidelines.

### Method details

#### Optomotor performance assay

The optomotor assay was designed to stimulate fish to swim back and forth across an arena using visual motion stimuli with systematically varied parameters. Fish were imaged at 200 Hz and 1 MP resolution using a Basler ace acA1920-155uc (Basler AG) USB3 camera equipped with an 8 mm SWIR fixed-focal-length lens and long-pass filter (Edmund Optics), with an imaging field of view of approximately 15 × 15 cm. Behavioral illumination was provided by four IR LED panels (850 nm; Autens IR Illuminator, Amazon). Visual stimuli were delivered by a DLP pico projector (P7, AAXA Technologies Inc.), with visible light reflected upward to the underside of the platform by a 25 × 20 cm cold mirror (Knight Optical), while allowing infrared illumination from below to pass through for camera imaging. Experiments were conducted in a circular glass Petri dish (VWR; 15 cm diameter, representing ∼22–33 body lengths for cichlids or zebrafish, respectively) filled to a height of 1 cm with filtered system water. The glass dish sat flush against a mylar screen upon which visual stimuli were projected from below.^67^ Fish were transferred from their home tanks into the dish using a plastic pipette. Separate dishes were used for zebrafish and cichlids.

Fish tracking and stimulus control were implemented using a custom workflow in BonZeb.^68^ Two stimulus update zones were defined at the leftmost and rightmost edges of the arena, extending inward up to 3.75 cm. When a fish entered one of these zones, the direction of stimulus motion reversed, such that entry into the left zone triggered rightward motion and vice versa. Three stimulus speeds (1, 2, and 3 cm/s) and three spatial periods (0.5, 1, and 2 cm) were tested, resulting in nine stimulus permutations. As the distance of the fish eye from the moving stimuli was not fixed, we used a distance of 0.5 cm, corresponding to the center of the water column. Cycles per degree were calculated with the formula 1/(2∗arctan∗[*W*_*cycle*_*/d*]), where *W*_*cycle*_ is the width of one cycle of alternating black and white bars and *d* is the viewing distance of 0.5 cm. Notably, due to water refraction creating Snell’s Window and the absence of any appreciable air gap below the dish, a 97° cone of space can be perceived. Each fish was presented with all permutations in a randomized order, with each condition tested twice, once in each direction, yielding a maximum of 18 trials per fish. Trials lasted up to 60 s. A trial was considered successful if the fish crossed to the opposite side of the arena within 60 s, at which point the stimulus parameters were updated. If the fish failed to cross within 60 s, the trial was marked as unsuccessful and the stimulus was updated with new parameters moving in the same direction. Because the starting position of the fish was unpredictable, the first stimulus presentation was excluded from trial counts. To focus analyses on animals that robustly performed the task, exclusion criteria were applied. Fish were excluded if they completed fewer than 50% of trials or spent more than 85% of the experiment duration within 2 cm of the dish edge.

#### Behavioral quantification and statistical analysis

Swimming gain, trial duration and swim direction were used to compare optomotor behavior across species. Fish centroid positions were smoothed by convolving changes in x and y displacement with a 100 ms boxcar filter to remove small tracking fluctuations. Swimming direction was calculated from the instantaneous change in centroid displacement during periods when swim velocity exceeded 0.5 cm/s, and data were pooled across fish within each species. Distributions were compared using two-sample Kolmogorov-Smirnov tests. Swimming gain was defined as the ratio of instantaneous swim velocity to stimulus velocity. Mean gain values were computed for each trial at each combination of stimulus speed and spatial period. A three-way mixed ANOVA was used to assess the effects of species (between-subject factor), stimulus speed (within-subject factor), and spatial period (within-subject factor). Tukey’s post hoc tests were used for pairwise comparisons. Trial duration was analyzed using the same statistical framework.

#### Optomotor task with continuous stimulation

To assess the effects of sustained optomotor stimulation, the same experimental setup was used with minor modifications. Only zebrafish and cichlids at 14 dpf were tested. In contrast to the back-and-forth task, stimulus parameters were held constant at a speed of 1 cm/s and a spatial period of 1 cm, and the stimulus was presented continuously for 15 min. To quantify thigmotaxis-like behavior, the time each fish spent within a circular zone located 2 cm from the arena wall was measured. A thigmotaxis score was calculated for each fish as the fraction of the 15 min trial spent within this zone. Thigmotaxis scores were compared across species using an independent-samples *t* test.

#### Optomotor task with varying bar widths

Inspired by Kist and Portugues (2019),^32^ a novel optomotor assay was developed using black-and-white gratings with randomly varying bar widths. The same hardware configuration described above was used. Zebrafish and cichlids at 14 dpf were tested. Stimuli moved at a constant speed of 1 cm/s for 5 min, with bar widths drawn from a uniform continuous distribution ranging from 0.25 to 2 cm. A new set of bars was generated at the start of each trial. Stimulus edges and bar polarity within the stimulation area were continuously recorded. To extract local stimulus features, the stimulus was first normalized to the fish centroid position and rotated to align with the fish’s forward-facing direction using the sign of the cosine heading angle. Because the offset between centroid tracking and eye position differed between species, species-specific eye offsets were determined from representative videos (∼1 mm for cichlids and ∼0.2 mm for zebrafish).

Local stimulus features were quantified within a region extending 2 cm in front of and behind the fish. Periods of robust optomotor behavior were identified using heuristic criteria based on swimming speed (>0.5 cm/s), alignment of swimming direction with the stimulus (within ±45°), and central positioning within the arena to avoid edge-related visual distortions. Due to species differences in swimming kinematics, continuous following periods were analyzed for cichlids, whereas pre-bout periods (10 ms before bout onset) were analyzed for zebrafish. Normalized stimulus features were averaged across all qualifying periods for each fish and then across all fish within a species. As a control, stimulus features were sampled at random time points, producing noise-like patterns, confirming that the structured features observed during follow and pre-bout periods reflected true local stimulus cues coupled to optomotor swimming.

#### Virtual open-loop assay for swim kinematics

A Sentech STC-CMB200PCL Camera Link camera (Aegis Electronic Group, Inc.), fitted with a Kowa LMZ45T3 zoom lens and long-pass filter, recorded video at 332 Hz and 1 MP resolution over an ∼2 × 2 cm field of view. Visual stimuli were delivered using a LightCrafter 4500 projector (EKB Technologies) at 120 Hz, coupled to a 120 W mercury light source (X-Cite Series 120), with visible light reflected to the underside of the platform using a cold mirror (Knight Optical). Fish were placed in a 10 cm diameter Petri dish filled to a height of 1 cm with filtered system water. Concentric circular gratings moving inward were used to guide fish into the field of view, at which point centroid position and heading direction tracking began. When fish crossed into a central circular zone (∼1 cm diameter), virtual open-loop stimulation commenced.

Four virtual open-loop conditions were tested, corresponding to forward, backward, leftward, and rightward perceived optic flow. In all conditions, optomotor gratings with a spatial period of 1 cm and a speed of 1 cm/s were continuously updated to remain fixed to the fish’s centroid and heading direction. Stimulation continued while the fish remained within a larger circular zone (∼1.5 cm diameter). When fish exited the 1.5 cm circle, concentric gratings were reintroduced to guide the fish back to the center. Each direction of optic flow was presented for 2 min, after which a new direction was randomly selected.

#### Kinematic tracking and analysis

Videos collected during virtual open-loop stimulation were tracked using DeepLabCut.^69^ A total of 84 zebrafish and 158 cichlid videos were analyzed. Separate DeepLabCut models were trained for each species. To account for the small size and low contrast of zebrafish pectoral fins, zebrafish videos were preprocessed using a custom background subtraction pipeline. Approximately 50 labeled images were used for training each model. For zebrafish, ∼80% of labeled images were selected from active swimming bouts to improve fin detection. Models tracked the tip of the mouth, swim bladder centroid, mid-tail point, tail tip, and the base and tip of both pectoral fins. Model accuracy was verified by comparing tracked points to manual annotations in 50 images per species, yielding errors of less than 3 pixels.

To assess temporal coordination across the fins and tail, pairwise cross-correlations were computed between the angular movement time series of the tail, left fin, and right fin (tail vs. left fin, tail vs. right fin, and right fin vs. left fin) and averaged across individuals within each species and optic flow condition (*n* = 4 per species; Figure 5). Cross-correlations were calculated using the circular cross-correlation function for finite discrete signals, defined as:

$$\left(f\;⋆\;g\right)\left[n\right]\;≜\sum_{m=0}^{N-1}\bar{f\left[m\right]}g{\left[\left(m+n\right)\right]}_{\mathrm{mod}\;N}$$

where *f* and *g* are the two joint angle time series being compared, *N* is the length of the sequence, and *n* is the lag index. For each pair of appendages, the cross-correlation was computed across a range of time lags, such that a lag of zero reflects the correlation between the two signals at the same moment in time, positive lags reflect the correlation of the leading signal with future values of the lagging signal, and negative lags reflect the correlation with past values. Each behavioral sequence was analyzed independently, yielding a distribution of correlation values at each lag. The mean and SEM of this distribution are reported.

Recordings used for cross-correlation analyses comprised 154 videos (520.2 s total) for *A*. *burtoni*: 28 videos (35.8 s) for caudal to rostral optic flow; 50 videos (180.6 s) for rostral to caudal optic flow; 27 videos (140.9 s) for leftward; and 49 videos (162.8 s) for rightward; and 66 videos (226.6 s total) for *D*. *rerio*: 18 videos (41.1 s) for caudal to rostral optic flow; 12 videos (45.9 s) for rostral to caudal optic flow; 27 videos (107.2 s) for leftward; and 9 videos (32.4 s) for rightward.

Swimming kinematics (Figure 6) were analyzed by pooling trials across individuals. Heading angular velocity was calculated by taking the temporal derivative of the heading angle, smoothing the resulting signal with a 30 ms boxcar filter, and converting values from degrees per frame to degrees per second. We first compared heading angular velocity using the full dataset for each species. Analyses were restricted to heading angular velocity values within the range of −200°/s to 200°/s to emphasize continuous, low-magnitude changes in heading direction observed in *A*. *burtoni*. Pectoral fin angle traces were used to compute interpeak intervals, fin amplitudes, and fin asymmetry, defined as the left minus right fin angle. All distributional comparisons were performed using two-sample Kolmogorov-Smirnov tests.

### Quantification and statistical analysis

All analyses were performed using custom scripts in Python and R. NumPy and Pandas were used for data processing and organization. Statistical analyses were performed using SciPy, Pingouin, and statsmodels. Jupyter notebooks were used to manage and document analysis workflows. See detailed experimental methods above for specific tests performed. Figures and trajectory plots were generated using Matplotlib and OpenCV. Asterisks in figures denote statistically significant differences across groups. See corresponding figure legends for statistical values and tests.

## Supplemental information

Document S1. Figures S1–S4

Video S6. D. rerio pose tracking during leftward optic flow Video shows a 14 dpf *D*. *rerio* performing the OMR during leftward optic flow. Video was acquired at 332 Hz with 1,088 x 1,088 pixel resolution. The background was subtracted from the video to improve contrast of the pectoral fins. Pose tracking of the pectoral fins and tail was performed using DeepLabCut post data acquisition

Video S7. *A*. *burtoni* pose tracking during leftward optic flow Video shows a 15 dpf *A*. *burtoni* performing the OMR during leftward optic flow. Video was acquired at 332 Hz with 1,088 x 1,088 pixel resolution. Pose tracking of the pectoral fins and tail was performed using DeepLabCut post data acquisition
