# Supplementary material for: Complex pectoral-fin-driven locomotion underlies refined visuomotor behavior in the cichlid Astatotilapia burtoni
Source: iScience. 2026 Aug 18;29(9):117136. doi: 10.1016/j.isci.2026.117136 (PMC13520728; doi:10.1016/j.isci.2026.117136)

iScience, Volume *29*

Supplemental information

Complex pectoral-fin-driven locomotion underlies refined visuomotor behavior in the cichlid *Astatotilapia burtoni*

Nicholas C. Guilbeault, Molly E. Westbrook, Venkatesh S. Krishna, Rida Ansari, Alexander Keth, Aristides B. Arrenberg, Scott A. Juntti, and Tod R. Thiele

Supplemental Figure 1. Optomotor performance is similar across assayed larval ages
A) Left: Mean swimming gain in 10-12 dpf and 13-15 dpf *A. burtoni*, collapsed across all performance assay trials. Right: Mean trial duration. Circles represent individual fish means. n = 18 (10–12 dpf), n = 31 (13–15 dpf).
B) Left: Mean swimming gain in 7 dpf and 14 dpf *D. rerio*, collapsed across all performance assay trials. Right: Mean trial duration. n = 7 (7 dpf), n = 11 (14 dpf).
Error bars represent mean ± standard deviation. *** $p < 0.05$ (independent samples t-test); n.s. = not significant.

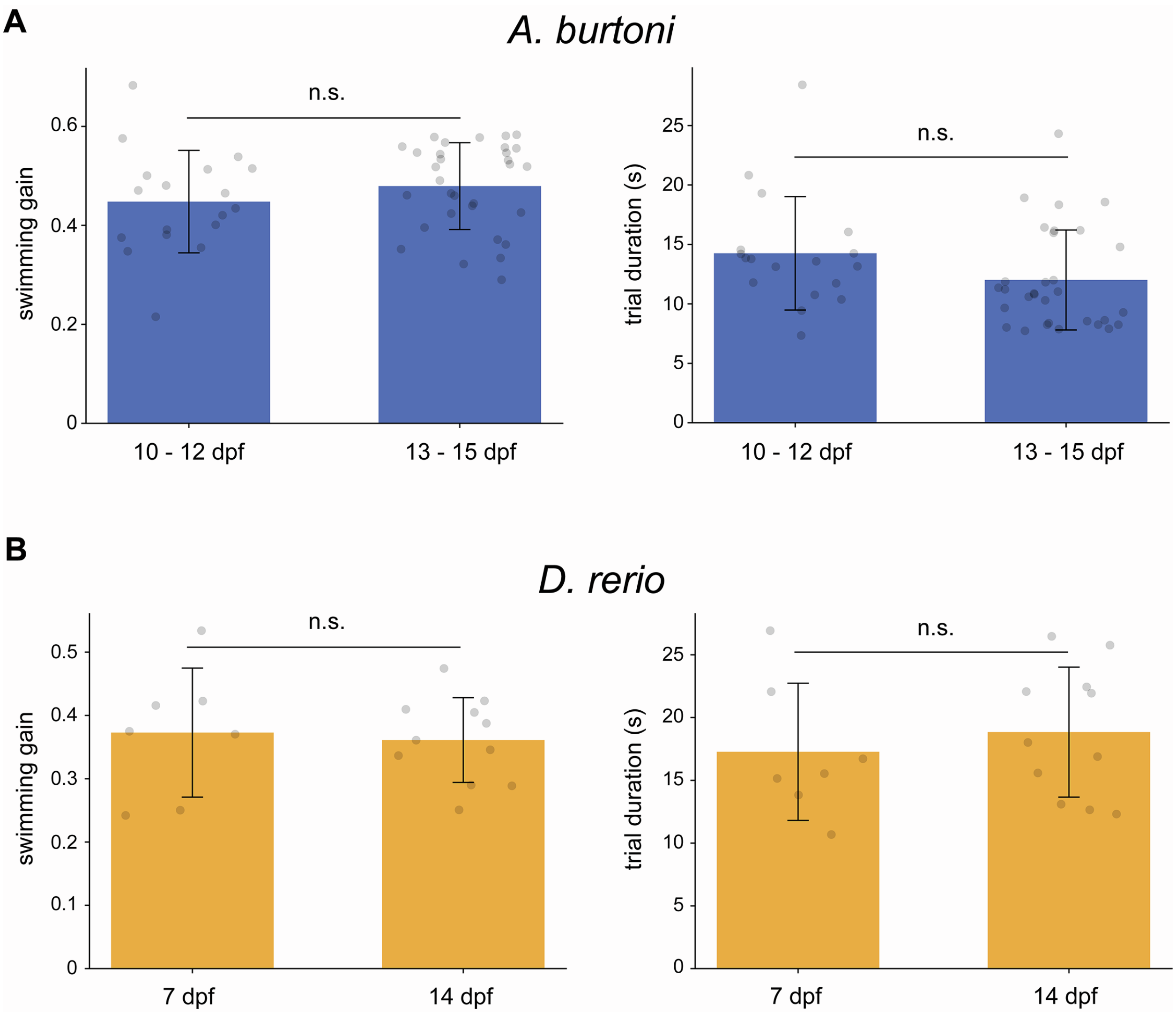

Supplemental Figure 2. Combined optomotor performance across all trials

A) Mean gain for each species generated by first averaging the gain of individual fish across all tested stimulus speed and spatial period combinations.

B) Mean trial duration for each species generated by first averaging the gain of individual fish across all tested stimulus speed and spatial period combinations.

A. burtoni n = 49; *D. rerio* n = 18. *** $p < 0.001$ (Independent samples t-test). Error bars represent mean ± standard deviation.

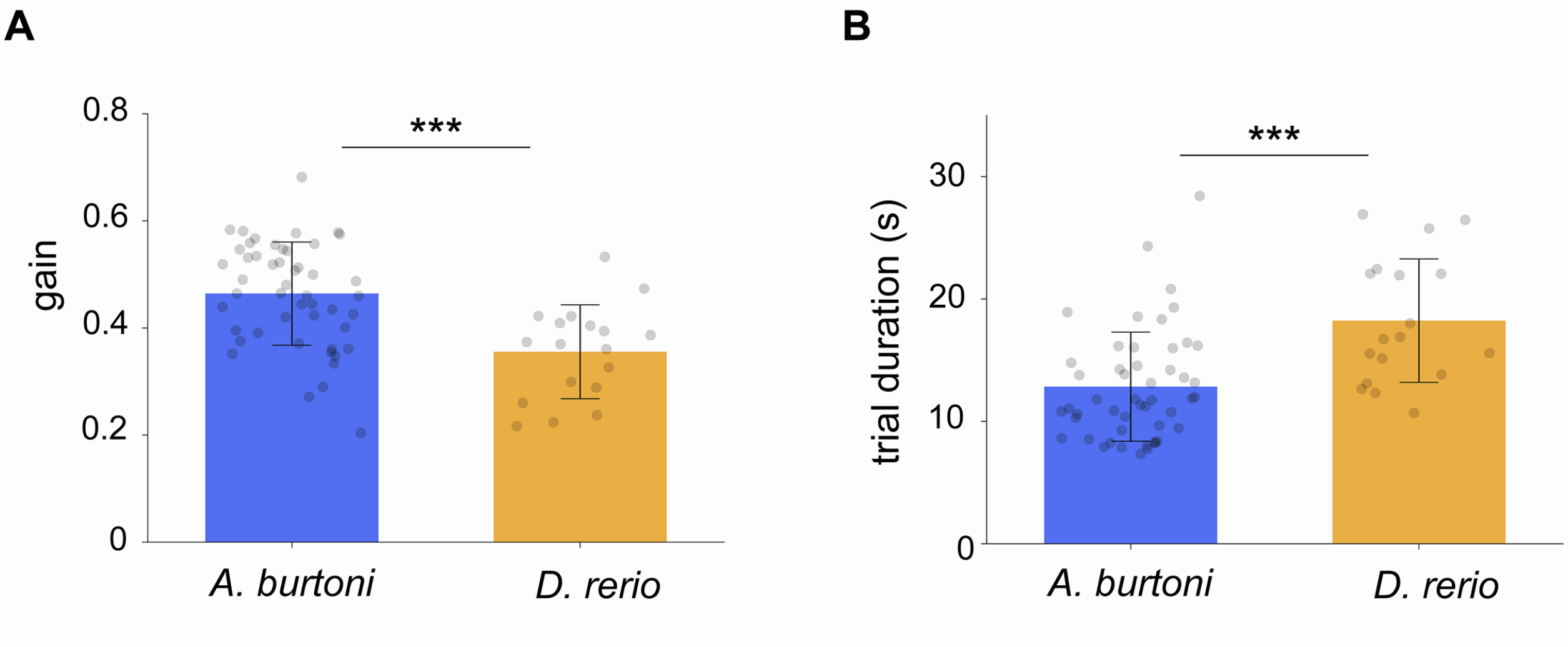

Supplemental Figure 3. *A. burtoni* engage in optomotor swimming for shorter duration than *D. rerio*

A) Left: Examples of an *A. burtoni* (blue) and *D. rerio* (yellow) trajectory inside the arena (red) during 15 minutes of continuous optomotor stimulation (1 cm/s, 1 cm bars). Boundary of the designated thigmotaxis zone is demarked by the circular dashed gray line. Middle: Fish position along the x-axis over time with respect to the midline of the arena (red). Changes in stimulus direction are shown as vertical dashed gray lines. Right: Fish distance from the center point of the arena over time. Shaded red regions indicate times when the fish swims within the thigmotaxis zone boundary (horizontal gray line).

B) Species comparison of thigmotaxis scores in the assay. *A. burtoni* n = 18; *D. rerio* n = 14. Error bars represent mean ± standard deviation.

C) Mean distance from arena center over time across all fish. Shaded region around the mean represents standard deviation. Dashed grey line represents the thigmotaxis zone boundary. *** $p < 0.001$ (independent samples t-test).

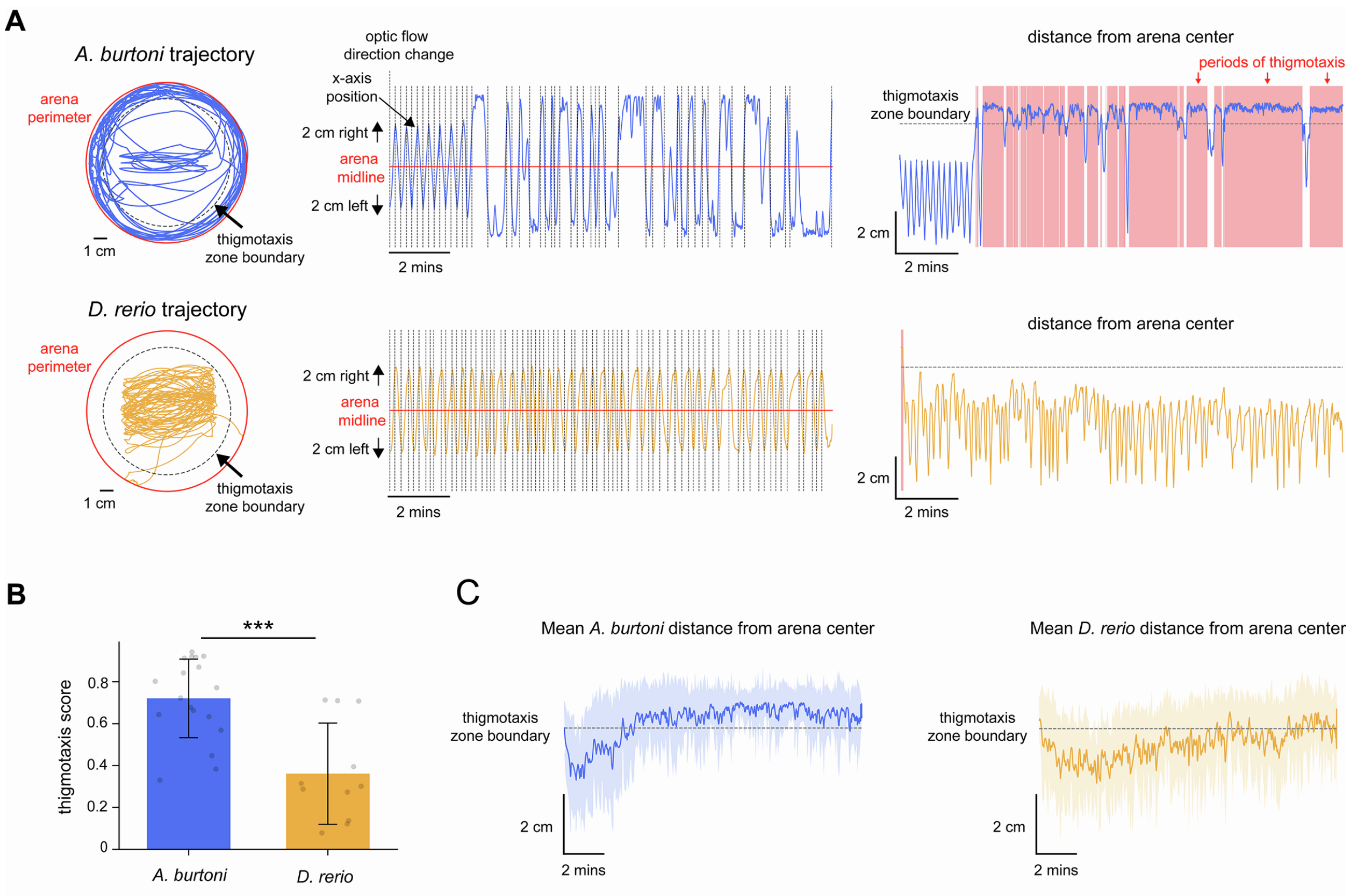

Supplemental Figure 4. *D. rerio* exhibit higher angular velocity heading changes
Angular velocity of heading direction was calculated by taking the temporal derivative of the heading angle. For visualization, values were log transformed to facilitate comparison of high-magnitude angular velocity events between species. *D. rerio* data were restricted to swim bouts, whereas the full dataset was used for *A. burtoni* due to its continuous swimming behavior. ***p < 0.001 (two-sample Kolmogorov–Smirnov test). *A. burtoni* n = 4; *D. rerio* n = 4.

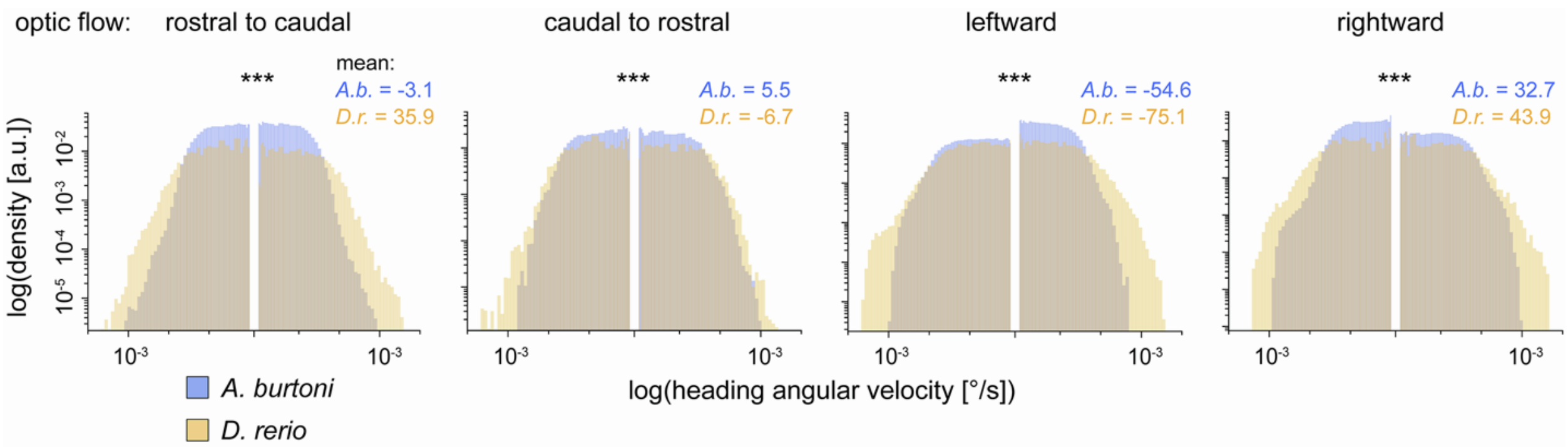

Supplement: Supplementary file 1 [file mmc1.pdf]
